# 

**Published:** 1898-05-07

**Authors:** 


					"The standard of highest purity,'"?lancet. Mat 7th, 1898. J
CAMY'S rfRJf CADJ?^YS ?
(~^ABSOLUTELY PURE. ?> ?J NO DRUGS OR ALKALIES USED.
No. 606; Vol. XXIV. PKSja Saturday,
May 7th, 1898.
?^NORFOLKamd NOR WICH HOSPITAL
[Bnbecriptkr^Terme,"j pRICB TWOPENCE.

				

